# BRCA1/2 NGS Somatic Testing in Clinical Practice: A Short Report

**DOI:** 10.3390/genes12121917

**Published:** 2021-11-28

**Authors:** Francesco Pepe, Pasquale Pisapia, Gianluca Russo, Mariantonia Nacchio, Pierlorenzo Pallante, Elena Vigliar, Carmine De Angelis, Luigi Insabato, Claudio Bellevicine, Sabino De Placido, Giancarlo Troncone, Umberto Malapelle

**Affiliations:** 1 Department of Public Health, University of Naples Federico II, 80131 Naples, Italy; francesco.pepe4@unina.it (F.P.); pasquale.pisapia@unina.it (P.P.); gianlucar93@libero.it (G.R.); mariantonia.nacchio@unina.it (M.N.); elena.vigliar@unina.it (E.V.); claudio.bellevicine@unina.it (C.B.); umberto.malapelle@unina.it (U.M.); 2 Institute for Experimental Endocrinology and Oncology (IEOS) “G. Salvatore”, National Research Council (CNR), 80131 Naples, Italy; plpallante@gmail.com; 3 Department of Clinical Medicine and Surgery, University of Naples Federico II, 80131 Naples, Italy; carmine.deangelis1@unina.it (C.D.A.); sabino.deplacido@unina.it (S.D.P.); 4 Department of Advanced Biomedical Sciences, School of Medicine, University of Naples Federico II, 80131 Naples, Italy; luigi.insabato@unina.it

**Keywords:** HGSOC, molecular pathology, *BRCA1/2*, PARPi, NGS

## Abstract

High-grade serous ovarian carcinoma (HGSOC) is the most common subtype of all ovarian carcinomas. HGSOC harboring *BRCA1*/*2* germline or somatic mutations are sensitive to the poly (adenosine diphosphate-ribose) polymerase inhibitors (PARPi). Therefore, detecting these mutations is crucial to identifying patients for PARPi-targeted treatment. In the clinical setting, next generation sequencing (NGS) has proven to be a reliable diagnostic approach *BRCA1/2* molecular evaluation. Here, we review the results of our *BRCA1/2* NGS analysis obtained in a year and a half of diagnostic routine practice. *BRCA1/2* molecular NGS records of HGSOC patients were retrieved from our institutional archive covering the period from January 2020 to September 2021. NGS analysis was performed on the Ion S5™ System (Thermo Fisher Scientific, Waltham, MA, USA) with the Oncomine™ BRCA Research Assay panel (Thermo Fisher Scientific). Variants were classified as pathogenic or likely pathogenic according to the guidelines of the American College of Medical Genetics and Genomics by using the inspection of Evidence-based Network for the Interpretation of Germline Mutant Alleles (ENIGMA) and ClinVar (NCBI) databases. Sixty-five HGSOC patient samples were successfully analyzed. Overall, 11 (16.9%) out of 65 cases harbored a pathogenic alteration in *BRCA1/2*, in particular, six *BRCA1* and five *BRCA2* pathogenic variations. This study confirms the efficiency and high sensitivity of NGS analysis in detecting *BRCA1/2* germline or somatic variations in patients with HGSOC.

## 1. Introduction

Ovarian cancer (OC) is the eighth most common cancer type among women worldwide and the leading cause of death for gynecological malignancies [1,2]. Morphologically, OCs are generally classified into Type I and Type II tumors. Whereas the former are generally low-grade and genetically stable tumors, the latter, which predominantly harbor Tumor Protein P53 (*TP53*) and Cyclin E1 (*CCNE1*) gene alterations, are more aggressive and genetically unstable [3]. Among Type II ovarian tumors, high-grade serous ovarian carcinoma (HGSOC) is the most common subtype, accounting for about three quarters of OCs [4,5]. In 96% of cases, HGSOCs carry *TP53* somatic mutations. However, in 22% of cases, they are associated with BRCA1 DNA Repair Associated (*BRCA1*) or BRCA2 DNA Repair Associated (*BRCA2*) germline or somatic gene mutations [6].

Unfortunately, OC remains asymptomatic for several years and goes undetected until it is advanced. Indeed, in about 70% of cases, the prognosis for HGSOC patients is rather bleak owing to late diagnosis [7]. Recently, considerable strides have been made in providing HGSOC patients with more effective personalized treatments, alongside traditional chemotherapy and antiangiogenic drugs.

Among the novel therapies, poly (adenosine diphosphate-ribose) polymerase inhibitors (PARPi) represent an important arrow in the oncologist’s quiver [8]. Indeed, PARPi have been shown to dramatically improve the clinical outcomes of HGSOC patients harboring *BRCA1*/*2* germline or somatic mutations [9,10,11,12]. Accordingly, current international guidelines widely recommend *BRCA1*/*2* testing in all patients with non-mucinous OC, including those with HGSOC [13,14,15]. Next generation sequencing (NGS) is emerging as a useful and popular tool for *BRCA1*/*2* testing in clinical practice thanks to its high sensitivity, ease of use, cost-effectiveness, and short turnaround time. Not surprisingly, our Molecular Predictive Pathology Laboratory at the Department of Public Health of the University of Naples Federico II routinely employs NGS to assess clinically relevant biomarkers in different solid tumors [16,17]. The clinical significance of this striking technology is reflected in the fact that since 2020, the Divisions of Oncology and Gynecology at our Institution have fully embraced the use of NGS in their routine clinical practice to screen patients for *BRCA1/2* germline or somatic mutations.

Here, we review our *BRCA1/2* NGS molecular results obtained during the last a year and a half of diagnostic routine practice.

## 2. Material and Methods

Records from previous *BRCA1/2* molecular tests carried out on HGSOC patients from January 2020 to September 2021 were retrieved from our internal archive. In particular, DNA extraction was performed with the QiAmp Mini Kit (Qiagen, Hilden, Germany) according to the manufacturer’s instructions. NGS analysis was performed on the Ion S5™ System (Thermo Fisher Scientific, Waltham, MA, USA) in combination with the Oncomine™ BRCA Research Assay panel (Thermo Fisher Scientific). This panel covers all the coding sequences in *BRCA1/2* genes, including all coding splice and acceptor sites, with an average of 64 bp extension into adjoining introns on Ion Torrent S5 (Thermo Fisher Scientific). In particular, library preparation and purification were manually performed according to the manufacturer’s instructions. A total of *n* = 8 amplified libraries were pooled together and diluted at 100 pM. Finally, template preparation and chip loading were performed automatically on the Ion Chef™ System (Thermo Fisher Scientific). Data inspection was carried out automatically by using the Ion Reporter Torrent Suite version 5.18.0.1 with a dedicated analysis workflow optimized for somatic annotation of *BRCA1/2* alterations. In detail, a minimum coverage of 500X, a quality score ≥20, and an allele mutation frequency of ≥5% were required to identify *BRCA1/2* mutations successfully. In addition, BAM files were visually inspected with the Golden Helix Genome Browser v.2.0.7 (Bozeman, MT, USA). Variant annotation was performed according to the Human Genome Variation Society nomenclature. Variants were classified as pathogenic or likely pathogenic (collectively termed pathogenic) according to the American College of Medical Genetics and Genomics (ACMG) recommendations by using the inspection of Evidence-based Network for the Interpretation of Germline Mutant Alleles (ENIGMA) and ClinVar (NCBI) databases.

## 3. Results

Overall, our in-house developed NGS workflow successfully analyzed a total of *n* = 65 HGSOC histological samples. Patients’ median age was 61.1 years (ranging from 25 to 91). All the histological samples were processed. In particular, the median value of neoplastic cell percentage was 59.7% (ranging from 10 to 90%). Nucleic acid isolation and quantification yielded a median value of 39.7 ng/µL (ranging from 0.6 to 60.0 ng/µL). As for the technical parameters, NGS analysis generated a median number of reads per sample of 1,382,380.2 (ranging from 505.0 to 13,533,583.0), a median number of read length of 106.5 bp (ranging from 101 to 122 bp), a median number of mapped reads of 1,370,850.3 (ranging from 505.0 to 13,391,178.00), a mean percentage of reads on target of 97.3% (ranging from 93.2 to 100%), an average of reads per amplicon of 6754.5 (ranging from 2.9 to 76,092.00), and a uniformity of coverage of 97.5% (ranging from 91.5 to 100.0%). Concerning the molecular results, whereas the vast majority of samples (54/65, 83.1%) showed no clinically relevant alterations, 11 (16.9%) out of 65 cases harbored a pathogenic alteration in *BRCA1/2*. In detail, six (54.5%) out of 11 mutated cases displayed a *BRCA1* pathogenic variation, whereas the remaining five (45.5%) harbored a *BRCA2* pathogenic alteration. Among the detected alterations, six (54.5%) were single nucleotide variants (SNVs) and five (45.5%) were small deletions or insertions. Moreover, one of the detected alterations was found in a non-coding region. Results are summarized in Table 1.

## 4. Discussion

The assessment of *BRCA1/2* molecular status has become part of the standard of care in the management of patients with HGSOC. Much progress has been made in the field of precision medicine against this type of cancer, which is responsible for over 60% of ovarian cancer-related deaths. A case in point is the development and clinical implementation of PARPi, which have been shown to improve the survival as well as quality of life patients affected by HGSOC. Thus, fast and reliable genetic screening for *BRCA1/2* germline or somatic mutations has become of paramount importance to identify patients who would most likely benefit from these therapeutic agents.

**Figure 1:**
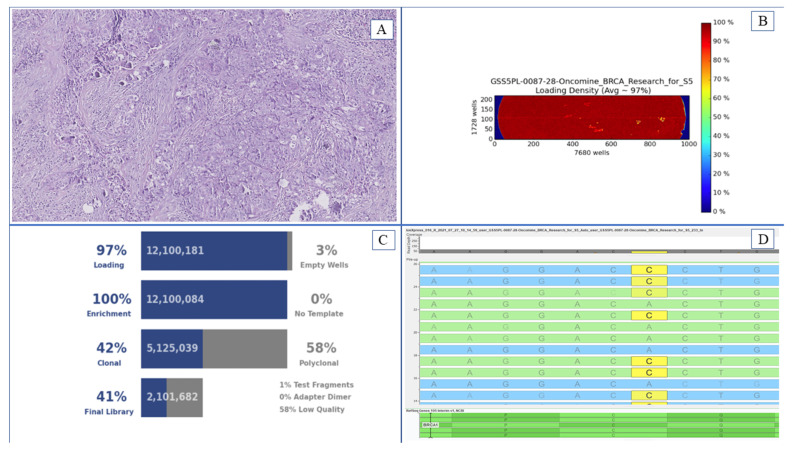
*BRCA1/2* molecular evaluation with NGS approach: an exemplificative case. This Figure also shows a neoplastic area for DNA extraction (**A**), loading density (**B**), and technical quality parameters (**C**) of a NGS run on an Ion Torrent S5 ™ platform (Thermo Fisher Scientifics). Box (**D**) shows a *BRCA1* p.C61G point mutation with an integrated genetics viewer.

This study highlights the high sensitivity, even in cases with a low neoplastic cells content, of NGS technology in detecting *BRCA1/2* pathogenic mutations in patients with HGSOC. In particular, our in-house developed NGS platform and workflow successfully evaluated the *BRCA1/2* status in a total of 65 HGSOCs. In line with previous published studies [6], our molecular analysis confirmed the presence of *BRCA1/2* pathogenic alterations in a substantial percentage (16.9%) of HGSOC patients. This strongly suggests the need to integrate *BRCA1/2* testing into routine clinical practice (Figure 1). In this setting, NGS, a robust and highly sensitive technology, provides clinicians with the opportunity to comprehensively evaluate *BRCA1/2* molecular status in both HGSOC and other types of cancer [18]. For over a decade now, NGS systems have revolutionized diagnostic practice by improving the success rates of molecular tests even when the diagnostic material is scant. Such paradigm-shifting technology has therefore laid the basis not only for an improved biomarker testing landscape but also for the development of multiple biomarker-based therapeutic strategies. With regard to *BRCA* genetic testing, our Predictive Molecular Pathology Laboratory at Federico II University Hospital regularly partakes in a national project to sensitize oncologists, primary pathologists, and molecular laboratories to the importance of *BRCA1/2* molecular analysis for HGSOC patients. To this end, the project has developed a dedicated website (http://www.brcafastnet.it, last access 16 Novermber 2021) able to oversee all clinical data exchange and shipment of biological material to all institutions involved in the project. Currently, a plethora of NGS panels are commercially available for *BRCA1/2* molecular testing. Despite the high heterogeneity in terms of technical approaches (e.g., chemistry, library preparations, and sequencing analysis) and data analysis (e.g., metrics and bioinformatics pipelines), several studies have long demonstrated a high degree of concordance among the variant cells [18].

In conclusion, we have presented a referral laboratory experience on *BRCA1/2* molecular analysis in unselected HGSOC patients from our diagnostic routine activity to highlight the crucial role of NGS analysis in the correct management of these patients. Further studies involving a larger gene panel are needed to investigate other promising gene alterations involved in homologous recombinant deficiency (HRD), which may expand the subset of HGSOC patients suitable for PARPi treatment.

## Figures and Tables

**Table 1:** Results obtained in our series of 65 high-grade serous ovarian carcinomas.

Patient	Age	Neoplastic Cells (%)	DNA Concentration (ng/µL)	Reads	Mapped Reads	Percent Read on Target (%)	Average Reads per Amplicon	Uniformity of Amplicon Coverage (%)	Mean Read Length (bp)	Molecular Result	Gene
1	74	70.00	19.90	1,103,756.00	1,100,608.00	98.15	3997.00	99.63	105.00	WT	
2	55	70.00	60.00	447,320.00	445,834.00	99.87	1615.00	98.90	104.00	WT	
3	51	60.00	41.00	990,443.00	987,075.00	99.87	2578.00	99.27	108.00	WT	
4	60	70.00	5.79	999,781.00	996,779.00	99.08	3617.00	98.53	104.00	WT	
5	42	70.00	60.00	1,225,736.00	1,221,759.00	99.04	4433.00	99.63	105.00	p.R1495M	*BRCA1*
6	66	70.00	2.41	940,118.00	937,071.00	98.71	3388.00	97.70	101.00	p.Q534X	*BRCA1*
7	69	70.00	25.40	1,134,001.00	1,129,619.00	98.79	4088.00	98.27	104.00	WT	
8	91	80.00	60.00	1,107,249.00	1,105,435.00	99.09	4012.00	98.90	105.00	WT	
9	72	70.00	60.00	978,740.00	977,513.00	98.93	3542.00	96.55	104.00	p.K830PfsTer18	*BRCA1*
10	66	70.00	60.00	1,015,943.00	1,014,574.00	99.33	3691.00	99.27	107.00	WT	
11	53	70.00	38.90	529,337.00	528,001.00	98.76	1910.00	99.63	106.00	WT	
12	71	50.00	25.30	1,111,471.00	1,109,964.00	99.28	4036.00	99.27	107.00	WT	
13	63	50.00	6.19	1,091,731.00	1,090,470.00	99.32	3967.00	98.53	106.00	WT	
14	61	70.00	60.00	1,120,367.00	1,118,600.00	99.15	4063.00	98.90	112.00	WT	
15	61	60.00	53.00	1,140,727.00	1,139,018.00	99.06	4133.00	98.99	110.00	WT	
16	25	50.00	60.00	1,052,429.00	1,051,081.00	99.06	3814.00	98.90	109.00	WT	
17	64	60.00	17.30	538,487.00	581,558.00	99.05	2110.00	99.63	109.00	WT	
18	68	80.00	60.00	574,298.00	572,675.00	98.75	2065.00	99.63	111.00	WT	
19	52	30.00	6.38	626,317.00	624,168.00	98.66	2256.00	99.63	115.00	WT	
20	69	70.00	60.00	667,821.00	665,818.00	98.91	2410.00	100.00	112.00	WT	
21	58	70.00	11.20	585,530.00	584,005.00	98.92	2116.00	99.63	112.00	p.Q 1756PfsTer74	*BRCA1*
22	59	80.00	60.00	516,539.00	514,737.00	98.62	1861.00	100.00	115.00	WT	
23	76	60.00	43.80	175,439.00	175,084.00	97.54	692.20	100.00	102.00	WT	
24	76	70.00	57.00	195,477.00	19,501.00	98.68	704.90	100.00	102.00	WT	
25	47	70.00	60.00	233,219.00	232,620.00	98.90	841.10	93.84	106.00	WT	
26	69	90.00	60.00	222,662.00	222,066.00	98.90	804.40	100.00	107.00	p.IVS2 + 1G > A	*BRCA2*
27	48	70.00	60.00	230,212.00	229,629.00	100.00	832.50	98.63	104.00	WT	
28	52	70.00	60.00	215,102.00	214,804.00	99.00	778.90	100.00	105.00	WT	
29	44	50.00	60.00	218,442.00	217,974.00	99.10	791.20	100.00	107.00	WT	
30	57	60.00	60.00	748,780.00	746,568.00	98.08	2682.00	100.00	106.00	WT	
31	45	50.00	32.20	633,164.00	631,252.00	98.12	2269.00	98.90	103.00	p.N319KfsTer8)	*BRCA2*
32	73	80.00	60.00	754,043.00	702,880.00	98.75	2542.00	100.00	105.00	WT	
33	63	60.00	60.00	808,451.00	806,608.00	98.57	2913.00	100.00	107.00	WT	
34	67	70.00	33.70	482,605.00	481,960.00	97.97	1727.00	96.30	103.00	WT	
35	39	80.00	51.00	932,611.00	931,119.00	97.67	3331.00	99.27	102.00	WT	
36	51	50.00	53.00	1,119,066.00	1,116,901.00	98.26	4020.00	100.00	105.00	WT	
37	66	70.00	7.15	1,020,993.00	1,019,060.00	98.89	3691.00	100.00	102.00	p.L1072Ter)	*BRCA2*
38	56	60.00	60.00	992,136.00	990,635.00	98.49	3574.00	100.00	102.00	WT	
39	57	70.00	60.00	1,952,836.00	1,949,593.00	98.64	7045.00	99.63	106.00	p.T1378Ter	*BRCA2*
40	77	70.00	60.00	1,584,808.00	1,582,139.00	98.47	5707.00	99.27	103.00	WT	
41	70	20.00	13.80	689,251.00	687,985.00	95.00	3914.00	94.61	104.00	WT	
42	60	50.00	60.00	668,595.00	667,463.00	94.00	3757.00	94.61	105.00	WT	
43	40	50.00	60.00	675,510.00	674,213.00	95.64	3861.00	95.21	104.00	WT	
44	70	50.00	22.20	505.00	505.00	97.03	2.90	91.57	105.00	RIP	
45	64	50.00	10.70	683,922.00	682,904.00	93.36	3815.00	95.21	102.00	WT	
46	52	10.00	51.00	619,053.00	617,877.00	94.31	3489.00	94.01	103.00	WT	
47	81	60.00	30.30	472,447.00	470,375.00	94.42	2654.00	93.41	109.00	WT	
48	67	70.00	60.00	204,918.00	204,318.00	93.72	1147.00	94.01	111.00	WT	
49	33	60.00	60.00	574,974.00	572,585.00	93.02	3189.00	95.21	109.00	p.Q1811Ter	*BRCA1*
50	72	70.00	60.00	13,533,583.00	13,391,178.00	94.89	76,092.00	95.81	105.00	WT	
51	74	40.00	5.40	9,481,551.00	9,408,339.00	94.13	53,031.00	95.21	104.00	WT	
52	49	50.00	19.70	12,876,898.00	12,766,795.00	94.17	71,989.00	96.41	103.00	WT	
53	48	60.00	60.00	13,327,661.00	13,176,126.00	94.45	74,523.00	96.41	106.00	WT	
54	73	60.00	60.00	1521.00	1503.00	93.35	8.40	93.53	103.00	WT	
55	53	60.00	18.80	592,625.00	590,434.00	94.04	3325.00	96.41	122.00	p.Q2157IfsTer18	*BRCA2*
56	76	20.00	0.60	459,548.00	458,573.00	96.02	2637.00	96.41	106.00	WT	
57	68	80.00	60.00	442,179.00	440,884.00	94.76	2502.00	95.81	108.00	WT	
58	62	50.00	8.50	382,324.00	381,454.00	96.15	2196.00	95.81	106.00	WT	
59	54	40.00	11.60	433,234.00	431,971.00	95.86	2480.00	95.25	111.00	p.C61G	*BRCA1*
60	66	70.00	47.00	407,607.00	406,640.00	95.03	2314.00	95.03	106.00	WT	
61	63	20.00	3.40	276,753.00	275,901.00	96.05	1587.00	95.21	111.00	WT	
62	62	70.00	37.80	280,573.00	279,771.00	96.01	1609.00	95.21	104.00	WT	
63	64	70.00	16.00	227,648.00	228,989.00	95.27	1295.00	94.01	107.00	WT	
64	74	40.00	4.14	275,446.00	274,321.00	95.03	1561.00	94.01	111.00	WT	
65	61	10.00	6.90	248,198.00	247,411.00	95.53	1415.00	95.21	112.00	WT	

## Data Availability

The data presented in this study are available on request from the corresponding author.
